# Mitochondrial cargo quality determines the paracrine effects of extracellular vesicles derived from vascular endothelial cells

**DOI:** 10.1016/j.biopha.2025.118751

**Published:** 2025-11-15

**Authors:** Zahid A. Manzar, Lucas J. Davis, Karl F. Swanson, Erik Muñoz, Hazel H. Szeto, Hans Minderman, B. Rita Alevriadou

**Affiliations:** a Vascular Mechanobiology Laboratory, Department of Biomedical Engineering and Center for Cell, Gene, and Tissue Engineering, University at Buffalo-SUNY, Buffalo, NY 14260, USA; b Social Profit Network, Menlo Park, CA 94025, USA; c Flow and Immune Analysis Shared Resource, Division of Medicine, Roswell Park Comprehensive Cancer Center, Buffalo, NY 14263, USA

**Keywords:** Vascular endothelial cell, Extracellular vesicles, Mitochondria, Mitochondrial transfer, Mitochondrial membrane potential, Inflammation

## Abstract

Cell-derived extracellular vesicles (EV) are mediators of intercellular communication with increased circulating levels of endothelial cell-derived EV (EC-EV) reported in cardiovascular diseases (CVD). The EC-EV ability to elicit either detrimental or restorative effects on target EC is thought to be, in part, due to horizontal transfer of their mitochondrial cargo. To understand the role of mitochondrial cargo in EC-EV paracrine effects, large EV were collected from media of cultured human EC, and the number of mitochondria-carrying EV (mitoEV), EV mitochondrial cargo mass, and mitoEV quality/polarization were quantified. EC activation with tumor necrosis factor (TNF)-α caused an increased release rate of EV (TNF-EV), including mitoEV that carried a larger and more depolarized mitochondrial cargo, compared to EV released from control EC (C-EV). EC co-treatment with TNF-α and the mitochondria-targeted antioxidant MitoTEMPO restored both the mitochondrial cargo quality and the number of mitoEV carrying polarized mitochondria to levels similar to C-EV. TNF-EV, but not C-EV, dose-dependently upregulated inflammatory gene expression in target naïve EC. Fluorescence microscopy showed the EV mitochondrial cargo to transfer and colocalize with the target EC mitochondrial network. Mitochondrial cargo depolarization of C-EV using carbonyl cyanide p-(trifluoromethoxy)phenylhydrazone was sufficient for those EV to trigger inflammation in target naïve EC. In conclusion, the mitochondrial redox state of donor EC regulates mitoEV mitochondrial cargo quality that, at least in part, determines their capacity to cause target EC dysfunction and promote CVD. The mitochondrial membrane potential (ΔΨ_m_) in EC-mitoEV may be a new biomarker and therapeutic target in vascular biology and medicine.

## 1. Introduction

Endothelial cell (EC) dysfunction, the first and most critical step in atherosclerosis development, is known to trigger release of EC-derived extracellular vesicles (EC-EV) [1–3]. Plasma levels of EC-EV have been found to be elevated in patients with hypertension, stable angina, coronary artery disease, acute coronary syndrome, and heart failure, and to correlate with adverse cardiovascular events suggesting that EC-EV may be prognostic and diagnostic biomarkers of atherosclerosis and cardiovascular disease (CVD) [4–8]. While EV are known vectors for intercellular transfer of proteins, nucleic acids, and lipids, increasing evidence suggests they also shuttle mitochondria [9–11]. Since most EC mitochondria have long axes between 500 nm-1.5 μm with an average of ~1 μm [12,13], whole mitochondria and/or mitochondrial fragments are more likely to be found carried inside of large-size EV (lEV, also called microparticles or microvesicles; defined as having diameters between 200 nm-1 μm [14]) compared to small-size EV (sEV, also called exosomes; defined as having diameters <150 nm [14]). Indeed, a significant percentage of lEV derived from EC, as well as various other cells, has been found to carry mitochondria [10,11,15]. In this study, the terms EV and mitoEV will be used to describe lEV and the mitochondria-carrying subset of lEV, respectively, and will refer to EC-EV, unless specified to be derived from other cells.

There is a dichotomy in the literature regarding the beneficial vs. harmful effects of EV, including mitoEV, transfer on recipient cell function: When EV from induced pluripotent stem cell-derived cardiomyocytes (iCM-EV) were applied to hypoxia-treated iCM, they improved the mitochondrial function and contractility of target cells, as well as left ventricular (LV) function in a mouse model of myocardial infarction [16]. Similarly, when EV from mesenchymal stem cells (MSC) were internalized by doxorubicin-treated CM, they inhibited reactive oxygen species (ROS) production and reduced the damage in target CM [11]. In the case of EC, the Manickam group showed the ability of EV derived from brain microvascular EC to improve mitochondrial function in recipient brain EC previously exposed to oxygen-glucose-deprived (OGD) conditions by increasing their ATP levels and oxygen consumption rate (OCR) [15,17]. The beneficial paracrine effect was abolished when OGD-EV were applied to OGD-EC suggesting the need for functional mitochondria in mitoEV from donor EC [15]. The beneficial effect was also absent when sEV derived from control EC were tested (possibly due to lack of mitochondrial cargo in sEV), and was decreased when EV were derived from other cell types suggesting a higher efficiency in homotypic EV mitochondrial transfer [15]. Despite the above beneficial effects of EV-mediated mitochondrial transfer, EV from either lipopolysaccharide (LPS)- or oligomycin-treated EC were shown to trigger inflammatory signaling in target naïve EC by increasing their mitochondrial ROS (mROS) production [18]. Similarly, EV from LPS-treated monocytes caused inflammation in target naïve EC; the effect was abolished when EV were derived from cells with impaired oxidative phosphorylation (ρ^0^) further supporting the role of EV mitochondrial cargo in paracrine effects [10].

Since EC inflammation/dysfunction, specifically EC mitochondrial dysfunction, is an early and critical step in atherosclerosis initiation and CVD development [19,20], it is important to better understand the mechanisms by which EV-mediated mitochondrial transfer causes EC injury. We hypothesized that two key determinants of EV-mediated EC inflammation are the poor quality of mitoEV mitochondrial cargo, characterized by loss of the mitochondrial membrane potential (ΔΨ_m_), and the concentration of mitoEV carrying depolarized/non-respiring mitochondria to target EC. By utilizing appropriate MitoTracker fluorescent probes and advanced imaging flow cytometry, we found that EV released from tumor necrosis factor (TNF)-α-treated EC (TNF-EV) carried a higher concentration of mitoEV with primarily depolarized mitochondria compared to EV released from control, untreated EC (C-EV). Both the mitochondrial cargo quality and the concentration of mitoEV with depolarized mitochondria were improved when donor EC were treated with TNF-α in the presence of the mitochondria-targeted antioxidant MitoTEMPO suggesting that the donor EC mitochondrial redox state determines these two mitoEV parameters. Upon incubation with target naïve EC, TNF-EV, but not C-EV, upregulated inflammatory gene expression in a dose-dependent manner. Flow cytometry and fluorescence microscopy confirmed the uptake and incorporation of mitoEV mitochondria to the donor EC mitochondrial network. To assess whether mitochondrial cargo quality, at least in part, determines the inflammatory paracrine effect, the mitochondria in C-EV were depolarized by C-EV treatment with the protonophore carbonyl cyanide p-(trifluoromethoxy)phenylhydrazone (FCCP), and their ability to cause inflammation in target naïve EC was examined. C-EV/FCCP, but not C-EV, were able to cause EC inflammation, although their capacity to do so was lower than that of TNF-EV. Our findings highlight the important role of the EC-EV mitochondrial cargo quality as a potential biomarker and therapeutic target in EV-mediated EC dysfunction and initiation of CVD.

## 2. Materials and methods

### 2.1. Reagents

Annexin V conjugated to fluorescein isothiocyanate (FITC; A13199), annexin V conjugated to allophycocyanin (APC; A35110), MitoTracker^™^ Green FM (M7514), MitoTracker^™^ Red FM (M22425), human TNF-α recombinant protein (A42551), DNase I solution, RNase-free (PI89836), and Invitrogen CellLight^™^ Mitochondria-red fluorescent protein (RFP) BacMam 2.0 (C10601) were purchased from ThermoFisher Scientific. Human red blood cell (RBC)-derived EV (CBS3) were from Cellarcus Biosciences. FCCP (SML-2959) and MitoTEMPO (SML0737) were from MilliporeSigma. Corning^™^ endothelial cell growth supplement (ECGS; CB-40006B), HyClone^™^ fetal bovine serum (FBS; SH3039602), and Lonza Walkersville gentamicin sulfate-amphotericin (GA-1000; NC0703289) were from Fisher Scientific. Heparin sodium salt from porcine intestinal mucosa (45-H3149) and L-glutamine (45–59202 C) were from Krackeler Scientific, Inc.

### 2.2. EC culture and treatments

Pooled primary human umbilical vein EC (HUVEC) were purchased from Lonza (C2519AS) and grown in complete EC growth medium from Lonza (EGM^™^ Endothelial Cell Growth Medium-2 BulletKit^™^; CC-3162) in a tissue culture incubator at 37 °C and an atmosphere of 5 % CO_2_/95 % air. EC of passage 3–8 were kept in T75 tissue culture flasks until they reached 90 % confluency. Upon confluency, EC were washed with phosphate buffered saline (PBS) and switched to a custom medium for EV collection composed of M199 basal media with Earle’s salts supplemented with 10 % FBS (depleted of pre-existing bovine EV), 2 mM L-glutamine, 100 μg/mL heparin, 30 μg/mL ECGS, 30 μg/mL gentamicin, and 15 ng/mL amphotericin. To deplete FBS of pre-existing bovine EV, FBS was diluted to 20 % v/v with M199 basal media and subjected to 100,000 g ultracentrifugation for 16 h at 4 °C. The bovine EV pellet was discarded, and the supernatant was used to make the EV collection media, as described above. EC kept in EV collection media for 24 h produced conditioned media containing control EV (C-EV). EC kept in EV collection media in the presence of TNF-α (10 ng/mL, 24 h) produced media containing TNF-EV. EC kept in EV collection media in the presence of MitoTEMPO (MT; 1 μM, 30 min preincubation followed by 24 h), at a concentration known to effectively scavenge superoxide radicals in activated EC [21–23], produced media containing C/MT-EV. Last, when EC were pretreated with MT (1 μM) for 30 min and TNF-α was introduced to the same media for 24 h, the conditioned media contained TNF/MT-EV.

### 2.3. EV isolation and characterization

#### 2.3.1. EV isolation from conditioned media

**Fig. 1.**
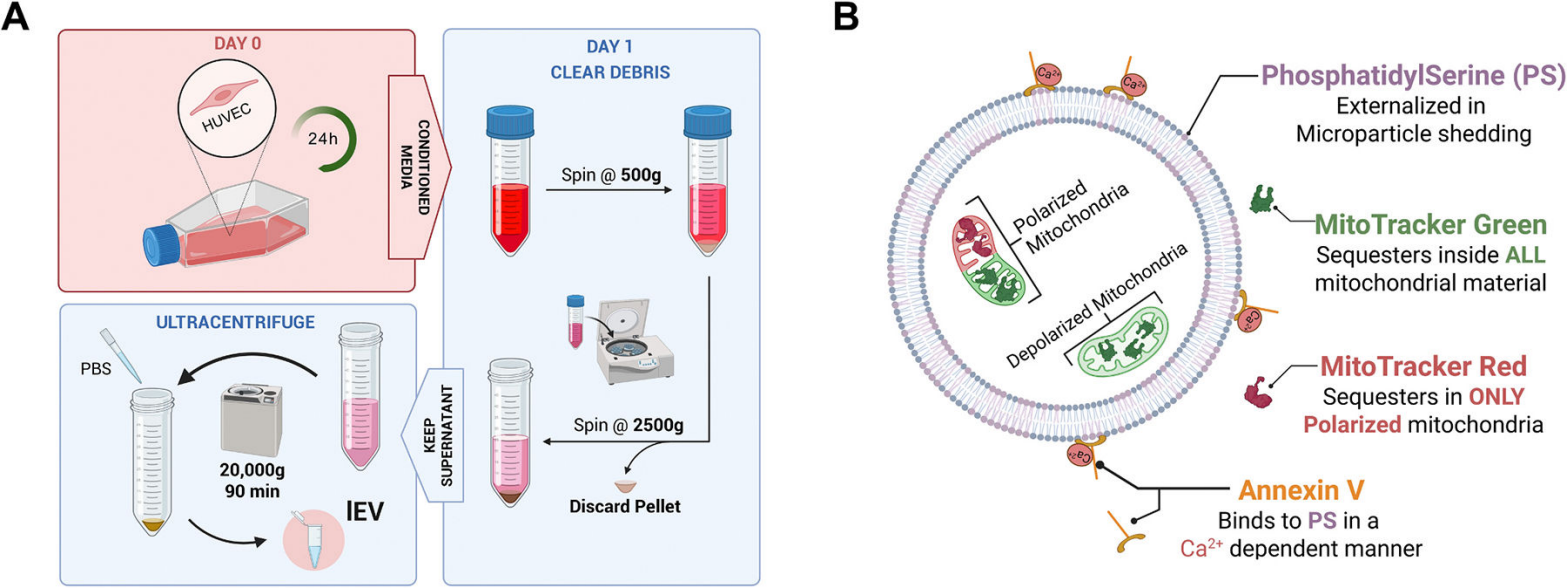
Schematic diagram of EC-EV isolation and staining protocol. (A) EV were isolated from conditioned media of cultured confluent early-passage HUVEC using differential centrifugation. Created in BioRender. Manzar, Z. (2025) https://biorender.com/uwalo0s. (B) Annexin V binds to PS on the EC-EV lipid membrane surface. EV were identified as Annexin V^+^ events. MitoTracker Green labels all the mitochondrial cargo inside mitoEV independently of ΔΨ_m_, whereas MitoTracker Red labels only polarized mitochondria inside mitoEV. Created in BioRender. Manzar, Z. (2025) https://biorender.com/6bn9tmx .

To isolate lEV, conditioned media was subjected to a differential centrifugation protocol (Fig. 1A). Briefly, the conditioned media was centrifuged at 500 g for 5 min at 4 °C to pellet cell debris. The supernatant was centrifuged at 2500 g for 10 min at 4 °C to pellet any apoptotic bodies. Finally, the supernatant was centrifuged at 20,000 g for 90 min at 4 °C resulting in the lEV pellet. The pellet was washed by resuspension in 1 mL of double-filtered chilled PBS in a 1.5 mL microcentrifuge tube, and subjected to a final spin at 20,000 g for 45 min at 4 °C. The final EV pellet was resuspended in the appropriate buffer depending on downstream experiments. EV were either used immediately or stored at 4 °C for no longer than overnight.

#### 2.3.2. EV size distribution measurement by nanoparticle tracking analysis (NTA)

For EV size distribution measurements, NTA was employed using a ZetaView analyzer (Particle Metrix) equipped with a 488 nm laser and complementary metal oxide semiconductor (CMOS) camera to track individual particle movements. The instrument was calibrated using 100 nm polystyrene standard beads. The final EV pellet was resuspended in PBS and further diluted to obtain concentrations of 100–200 particles/frame. For each size distribution measurement, two cycles were performed by scanning 11 cell positions/cycle and capturing 60 frames/position using the following settings: Sensitivity 70, shutter 100, scattering intensity 11.64, frame rate 7.5, and temperature 25 °C. Video frame analysis was performed by ZetaView software 8.05.12 that employs the Stokes-Einstein equation to convert the Brownian motion of each particle into its hydrodynamic diameter and generate a size distribution profile for each sample using the following parameters: Maximum particle area 5000, minimum particle area 20, minimum particle brightness 20, and minimum trace length 30.

#### 2.3.3. EV characterization and quantification by imaging flow cytometry

Since phosphatidylserine (PS) is known to be exposed on the outer leaflet of the EV lipid bilayer [24], Annexin V conjugated to FITC (or, in some experiments, APC) was used to fluorescently label the EV surface. Annexin V^+^ events were acquired and analyzed using a high-resolution imaging flow cytometric approach (Fig. 1B). Specifically, acquisition was performed on an Amnis ImageStream^X^ MkII-401 using INSPIRE software and settings of 40x objective, low flow rate/high sensitivity, and all lasers (405, 488, 561, 640, and 785 nm) activated at full voltage. The instrument utilizes 1 μm reference “speed beads” for internal calibration of the machine’s fluidics and image capturing system. Conditioned media from two T75 flasks was pooled and processed to generate one EV pellet that was resuspended in 1 mL of 1X Annexin binding buffer (140 mM NaCl, 2.5 mM CaCl_2_, 10 mM HEPES). 100 μL of that solution was diluted 1:8 v/v in binding buffer, and 20 μL of the diluted EC suspension was bought up to 60 μL final volume with binding buffer and stained with Annexin V-FITC (1:600 v/v, as determined by dye titration experiments) for 30 min on ice in the dark. Volumetric-based acquisition was used to determine EV concentration by measuring objects/mL of Annexin V^+^ events. To show EV degradation by Triton X-100, a similarly processed EV sample was incubated with 1 % Triton X-100 for 30 min prior to staining. Since Annexin V binding to PS is Ca^2+^-dependent, another EV pellet was resuspended in EDTA buffer (140 mM NaCl, 10 mM EDTA, 10 mM HEPES) and stained as before, to create an Annexin V^−^ control. Buffer-only and dye-only control samples were acquired to ensure clean sample preparation. At least 10,000 events were recorded and analyzed using the manufacturer’s IDEAS software version 6.2, and data was displayed on a scatter plot of FITC (channel 02) fluorescence intensity against side scatter (channel 12) intensity. Single color controls were acquired to assemble compensation matrices using INSPIRE software. Final compensation matrices and an analysis template were applied, using the batch processing feature, to the .rif files of each EV sample. The resulting .daf files were analyzed using IDEAS software version 6.2.

#### 2.3.4. MitoEV characterization and quantification by imaging flow cytometry

To detect the presence of mitoEV, EV samples were stained with both Annexin V-APC and MitoTracker Green (the latter accumulates in the mitochondrial matrix independently of ΔΨ_m_, and, hence, stains both respiring and non-respiring mitochondria [25]) and were analyzed at the Imagestream^X^ (Fig. 1B). Specifically, 20 μL of the diluted EV suspension was brought up to a final volume of 60 μL, and stained with Annexin V-APC (1:600 v/v) and MitoTracker Green (275 nM final concentration, as determined by dye titration experiments) for 30 min on ice in the dark. RBC-derived EV (known to be devoid of mitochondria) were stained in parallel and used to determine the background MitoTracker Green fluorescence [26,27]. Samples resuspended in EDTA buffer were used to determine Annexin V gating. Acquisition was performed on the ImageStream^X^ and settings were as follows: Brightfield (channels 01 and 09), MitoTracker Green (channel 02), side scatter (channel 06), and Annexin V-APC (channel 11). Acquisition was run for at least 2 min to record 10,000 events and data were displayed on a scatter plot of MitoTracker Green fluorescence intensity against Annexin V-APC fluorescence intensity. Instrument settings were changed to the 60x objective for acquiring images of every event/particle providing a brightfield pixel size of 0.3 μm by 0.3 μm, and used to extract individual particle size/area information in two EV subpopulations, mitoEV vs. EV devoid of mitochondrial cargo (dEV).

#### 2.3.5. MitoEV mitochondrial cargo polarization assessment by imaging flow cytometry

To assess the quality/polarization of the mitochondrial cargo in mitoEV, EV samples were stained with both Annexin V-FITC and MitoTracker Red (the latter stains only polarized mitochondria, because its accumulation in the mitochondrial matrix depends on an intact ΔΨ_m_ [25]) and analyzed at the Imagestream^X^ (Fig. 1B). MitoTracker Red median fluorescence intensity (MFI) was used to quantify the mitoEV mitochondrial cargo polarization state. 20 μL of diluted EV suspension was brought up to 57 μl with binding buffer, stained with Annexin V-FITC (1:600 v/v), and incubated for 30 min on ice in the dark. 3 μL of MitoTracker Red stock solution (100 nM final concentration) with or without FCCP (2.5 μM final concentration) was added and incubated for an additional 5 min followed by acquisition on the ImageStream^X^. Software was configured as follows: Brightfield (channels 01 and 09), Annexin V-FITC (channel 02), MitoTracker Red (channel 05), and side scatter (channel 12). Acquisition was run for at least 2 min to record 10, 000 events and only Annexin V^+^ events were displayed on a histogram of MitoTracker Red fluorescence intensity. For each experiment, the percentage of polarized mitoEV was defined by setting a threshold on MitoTracker Red intensity. The threshold was determined using FCCP (2.5 μM, 5 min) to achieve almost complete depolarization of C-EV (as determined by titration experiments) and selecting 5 % of EV with the highest MitoTracker Red signal intensity.

#### 2.3.6. EV analysis by transmission electron microscopy (TEM)

EV suspensions were pelleted at 20,000 g in an Eppendorf 5424 R centrifuge for 45 min. EV pellets were fixed in 4 % paraformaldehyde and 2.5 % glutaraldehyde in 0.1 M sodium cacodylate for a minimum of 24 h. EV pellets were washed twice in cacodylate buffer and post-fixed in 1 % OsO_4_, 1.5 % K_3_Fe(CN)_6_ for 1 h. Following washes in double-distilled H_2_O, the pellet was trapped in 3 % agarose. The EV pellet was then processed through dehydration using a graded series of 50–100 % ethanol. Samples were infiltrated with a propylene oxide Embed 812 resin mixture, embedded in Embed 812 resin, and polymerized for 48 h at 65 °C. One μm semi-thin sections were used to target regions of interest for ultrathin (70 nm) sections which were collected on slot copper grids and stained with 2 % uranyl acetate followed by 0.3 % lead citrate for 5 min each. Ultrathin sections were imaged using a Hitachi 7650 transmission electron microscope (Hitachi America) at 80 kV fitted with a side mount AMT NanoSprint12 digital camera (Advanced Microscopy Techniques).

### 2.4. EV DNA extraction and quantitative polymerase chain reaction (qPCR)

Conditioned media from control EC was split in half and processed to yield EV pellets. The first EV pellet was used to quantify nuclear DNA (nucDNA) and mitochondrial DNA (mtDNA) present only inside the EV. Briefly, the EV pellet was treated with 50 U/mL DNase I for 1 h at 37 °C and then DNase I was inactivated with 50 mM EDTA for 10 min at 65 °C. To lyse the EV, 1x RIPA buffer was added and incubated for 30 min on ice. The second EV pellet did not undergo DNase I treatment and was directly lysed with 1x RIPA buffer. Both samples were processed with DNeasy Blood and Tissue Kit (from Qiagen; 69504) to isolate DNA that was eluted into nuclease-free H_2_O. Following DNA isolation, qPCR was performed in triplicate using the iTaq Universal SYBR Green Supermix (from Bio-Rad; 1725120) and CFX Connect Real-Time PCR Detection System (from Bio-Rad; 1855201). The PCR program consisted of an initial step at 95 °C for 30 s, followed by 40 cycles of denaturation at 95 °C for 3 s, annealing at 60 °C for 20 s, followed by melting at a gradient from 65 to 95 °C. Normalized fold expression was calculated and plotted using the ΔΔCt method. Primer sequences to detect the nuclear gene β–2-microglobulin (B2M) and mitochondrial genes cytochrome b (CYTB), mitochondrial tRNA-Leu (UUA/G) 1 (MT-TL1), and NADH dehydrogenase 1 (ND1) [28] were verified for specificity using Primer-BLAST software. These sequences were B2M: 5′-CCACTTCATCCACGTTCACC-3′ and 5′-GAAGAGCCAAGGACAGGTAC-3′, CYTB: 5′-ATCACTCGAGACGTAAATTATGGCT-3′ and 5′-TGAACTAGGTCTGTCCCAATGTATG-3′, MT-TL1: 5′-CCTCGGAGCAGAACCCAACCT-3′ and 5′-CGAAGGGTTGTAGTAGCCCGT-3′, and ND1: 5′-CGAAAGGACAAGAGAAATAAGC-3′ and 5′-CTGTAAAGTTTTAAGTTTTATGCG-3′.

### 2.5. MitoEV mitochondrial cargo uptake assessment

#### 2.5.1. Qualitative uptake assessment by fluorescence microscopy

Confluent donor EC in T75 flasks were stained with MitoTracker Green (100 nM) for 30 min, washed three times with PBS, and maintained in EV collection media for 24 h. Conditioned media was processed to isolate EV, and the mitoEV subset was confirmed to carry MitoTracker Green-stained mitochondria on the ImageStream^X^. A day prior to EV isolation, target naïve EC had their mitochondria labeled using the baculovirus CellLight^™^ Mitochondria-RFP BacMam 2.0, a fusion construct of the E1 alpha pyruvate dehydrogenase leader sequence and monomeric RFP for mitochondrial targeting. Target EC were transduced according to the manufacturer’s protocol: Briefly, EC of passage 3–5 were seeded on plastic parallel-plate flow chamber slides with high optical quality (μSlide 0.6 luer with 1.5 mm coverslip from ibidi; 80186) at a density of 40,000 cells/cm^2^. EC reached 60 % confluency in 24 h and were then incubated with the viral vector at a multiplicity of infection (MOI) of 40 for 6 h. Media was changed following the 6 h transduction. EC reached confluency 24 h later and were then incubated with EV (with stained mitoEV mitochondria) at doses corresponding to EV:EC ratios of either 75 or 300 in complete M199 medium. Fluorescence microscopy images of target EC were captured at 63x magnification using LAS X life science microscope software followed by Thunder 3D imaging (Leica). Z-stack maximum projection, and XY and YZ projections were constructed using fluorescence images taken at a 0.4 μm step covering the thickness of the EC mitochondrial network, which is ~5 μm, as shown in our prior work [12], and used to detect colocalization of green and red signals.

#### 2.5.2. Quantitative uptake assessment by flow cytometry

Flow cytometry was employed as an orthogonal approach to quantify mitoEV mitochondrial cargo uptake by target EC. Similar to the protocol described above, EV were isolated from donor control EC stained with MitoTracker Green. Unlabeled target EC were grown to confluency in 96-well plates and treated with EV for 24 h, at same doses as described above, in complete M199 medium. Following EV treatment, target EC were analyzed at a LSRFortessa^™^ X-20 flow cytometer (Becton Dickinson). EC were gated for forward and side scatter, and only singlet events were assessed for MitoTracker Green signature indicating uptake of the mitoEV mitochondrial cargo. Threshold gating was determined using EC labeled with MitoTracker Green (positive control) and EC neither labeled with MitoTracker Green nor treated with mitoEV (negative control).

### 2.6. Target EC RNA extraction, reverse transcription, and qPCR

Total RNA was extracted from EC at the end of 24h incubation with either EV or vehicle using the RNeasy mini kit from Qiagen (74104). Reverse transcription was performed using the iScript cDNA synthesis kit from Bio-Rad (1708890). qPCR was performed in duplicate using the iTaq Universal SYBR Green Supermix from Bio-Rad (1725120) and the CFX Connect Real-Time PCR Detection System from Bio-Rad (1855201). Expression of inflammatory genes intercellular adhesion molecule (ICAM)-1, vascular cell adhesion molecule (VCAM)-1, monocyte chemoattractant protein (MCP)-1, and interleukin (IL)-8, known to be upregulated in TNF-α-treated EC [29–31], was normalized to that of housekeeping genes glyceraldehyde-3-phosphate dehydrogenase (GAPDH) and β-actin (ACTB). Normalized fold expression was calculated and plotted using the ΔΔCt method. Primer sequences were verified for specificity using Primer-BLAST software. These sequences were ICAM-1: 5′-TTGCTCAGGCAGAAATGGAC-3′ and 5′-AGGCAAACAGGTGTTCCTTCT-3′, VCAM-1: 5′-ACTGTGATGGCAATGGCGAA-3′ and 5′-TAAAGCGAAGTCCCAGGCAG-3′, MCP-1: 5′-AGAGACCTTGGCGATAAAGGG-3′ and 5′-ATCCAGAATGGGGTTTAGTGAGG-3′, IL-8: 5′-AGAAGCTCATTCGGAAGCCAA-3′ and 5′-CCAGGAGTACACCCACCAC-3′, GAPDH: 5′-CCACAGTCCATGCCATCAC-3′ and 5′-CCACCACCCTGTTGCTGTA-3′, and ACTB: 5′-CCAACCGCGAGAAGATGA-3′ and 5′-CCAGAGGCGTACAGGGATAG-3′.

### 2.7. Statistical analysis

Welch’s *t*-test, a modified Student’s *t*-test recommended for small size samples that lifts the assumption of the standard Student’s *t*-test that the variances of two groups are equal, was used to compare the means of two groups. Statistically significant differences among the means of three or more groups were determined using one-way analysis of variance (ANOVA) followed by Tukey’s multiple comparisons post hoc test. Statistical symbols were as follows: *, P ≤ 0.05; **, P ≤ 0.01; ***, P ≤ 0.001; and ****, P ≤ 0.0001. Statistical analyses were performed using GraphPad Prism 9 software on a PC.

## 3. Results

### 3.1. A significant proportion of EC-EV are mitoEV

**Fig. 2.**
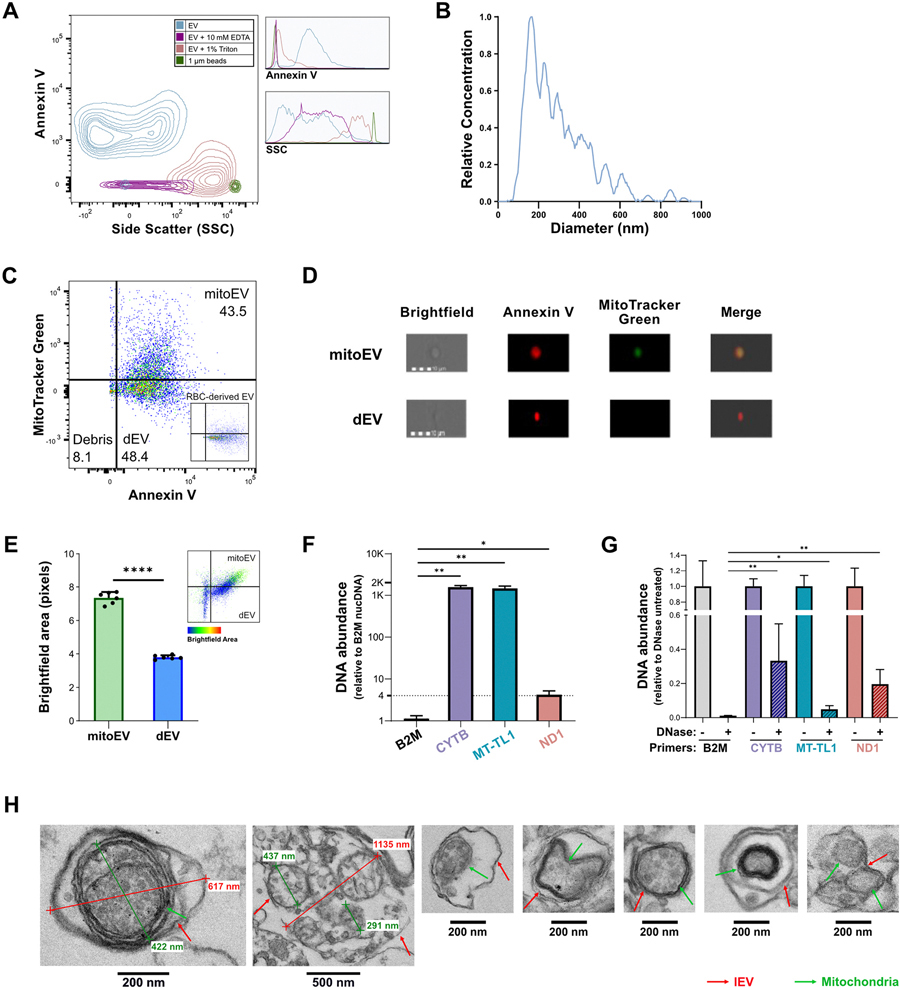
Characterization of EC-EV and their mitoEV subset. (A) Side scatter (SSC) plot shows Annexin V^+^ events that were abolished in the presence of either EDTA or Triton X-100. 1 μm reference beads were included for relative particle sizing. (B) A characteristic EC-EV size distribution (in nm) acquired by NTA is shown. (C) Using MitoTracker Green, EV were categorized based on whether they carry a mitochondrial cargo (mitoEV) or are devoid of it (dEV). RBC-EV were used to determine the background MitoTracker Green fluorescence. (D) Representative images (60x) acquired by ImageStream^X^ confirmed the presence of MitoTracker Green fluorescence (mitochondrial cargo) inside mitoEV and its absence inside dEV. (E) EV area information (in pixels) was extracted from brightfield images acquired by the ImageStream^X^, and plotted as mean±SE from n = 6 independent experiments. ****, P ≤ 0.0001. The subplot shows graphically the difference in size between mitoEV and dEV from a characteristic EV sample. (F) qPCR was used to measure expression of a nuclear gene (B2M) and three mtDNA genes (CYTB, MT-TL1, and ND1) in EC-EV. (G) MtDNA associated with EC-EV was more resistant to DNase I treatment than nucDNA suggesting that mtDNA is preferentially packaged inside the EV. Data are plotted as mean±SE of n = 3 independent experiments. *, P ≤ 0.05; **, P ≤ 0.01 compared to B2M expression in EV either without or with DNase I treatment. (H) Representative TEM images of sectioned EC-EV. Each image shows a lEV with diameter between 200 nm-1 μm (red arrows) carrying either a single or multiple mitochondria. Encapsulated mitochondria are shown as dense structures surrounded by a double membrane with diameters between 200 and 450 nm (green arrows).

EV isolated from media of control EC (C-EV) were characterized using imaging flow cytometry. C-EV were identified as Annexin V^+^ and degraded in the presence of Triton X-100 (Fig. 2A). Since Annexin V binding is Ca^2+^-dependent, EDTA was used to account for background fluorescence (Fig. 2A). Although EV were shown to lie in proximity to 1 μm reference beads (Fig. 2A), NTA was used to accurately measure EV size: EV were found to have a size distribution range of 100 nm-1 μm with an average diameter of ~200 nm, and, hence, the majority of them are lEV (Fig. 2B). EV labeling with both Annexin V and MitoTracker Green followed by imaging flow cytometry revealed two subpopulations: mitoEV (Annexin V^+^/MitoTracker Green^+^) that carry mitochondrial cargo, and dEV (Annexin V^+^/MitoTracker Green^−^) that are devoid of mitochondrial cargo; each comprised about half of total EV (Fig. 2C). MitoTracker Green-stained RBC-derived EV were used to determine the background MitoTracker Green fluorescence (Fig. 2C insert). Since the ImageStream^X^ takes fluorescence images of each event/particle, the spatial localization of each dye in a particle can be visualized. Visualization confirmed that Annexin V uniformly stained the surface of both mitoEV and dEV, whereas MitoTracker Green stained only the interior of mitoEV, where the mitochondrial cargo is located (Fig. 2D). When the pixel size of each event was measured from brightfield images, mitoEV were found to be, on average, significantly larger than dEV, probably due to their mitochondrial cargo (Fig. 2E). To further confirm the presence of mitochondria within mitoEV, total DNA was extracted from lysed EV isolate and the relative abundance of nucDNA vs. mtDNA was compared using primers specific for nuclear (B2M) and mitochondrial genes (CYTB, MT-TL1, ND1). Every mtDNA gene was found to be significantly more abundant than nucDNA suggesting that EV are selectively enriched in mtDNA (Fig. 2F). Notably, mtDNA was still present, whereas nucDNA was undetectable, following EV DNase I treatment suggesting that mtDNA is packaged inside EV and not simply adhered onto their surface (Fig. 2G). TEM images of sectioned EV provided the best evidence of the existence of mitoEV in EV samples, as well as information on the heterogeneity of mitoEV size and structure: MitoEV as small as 200 nm were found to contain a single mitochondrion, whereas the largest EV (~1 μm in diameter) carried multiple mitochondria (Fig. 2H). The cross sections of mitochondria inside mitoEV had typical lengths between 200 and 450 nm (Fig. 2H). Taken together, this data showed that control EC release mitochondria packaged inside mitoEV, which constitute about half of total EV.

### 3.2. mitoEV exhibit a functional proton gradient that can be inhibited by FCCP

**Fig. 3.**
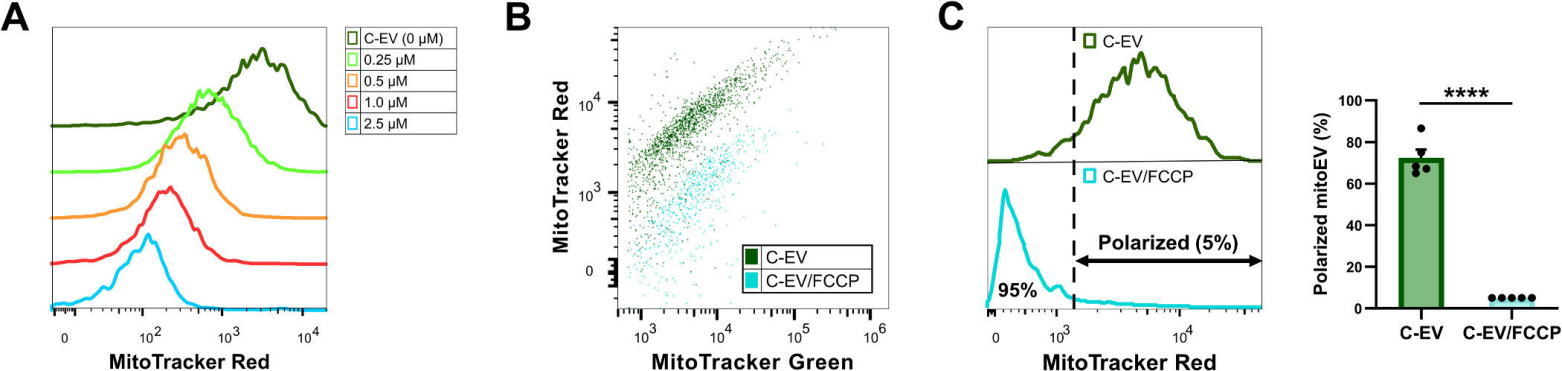
C-mitoEV are susceptible to mitochondrial cargo depolarization by FCCP. (A) FCCP dose-dependently depolarized C-mitoEV mitochondrial cargo as shown by a leftward shift in MitoTracker Red fluorescence. (B) FCCP treatment (2.5 μM, 5 min) of C-EV decreased the mitoEV mitochondrial cargo polarization (MitoTracker Red signal), but not the mitochondrial cargo mass (MitoTracker Green signal). (C) MitoEV were defined as carrying polarized cargo based on an MFI threshold that excludes 95 % of C-EV treated with FCCP (2.5 μM, 5 min) as fully depolarized. Data are plotted as mean±SE of n = 5 independent experiments. ****, P ≤ 0.0001 compared to C-EV.

C-EV were treated, or not, with the protonophore FCCP (disrupts the proton gradient across the mitochondrial inner membrane) followed by staining with MitoTracker Red and analyzed using imaging flow cytometry. Untreated C-EV stained strongly with MitoTracker Red (Fig. 3A). FCCP treatment (0.25–2.5 μM, 5 min) caused a dose-dependent depolarization of the C-EV mitochondrial cargo, as indicated by the leftward shift in MitoTracker Red intensity (Fig. 3A). When EV were double stained with MitoTracker Green and MitoTracker Red and then treated with FCCP (2.5 μM, 5 min), only the MitoTracker Red signal was lost suggesting that FCCP specifically depolarizes the mitochondrial cargo without affecting its mass (Fig. 3B). For gating of mitoEV with polarized mitochondria (polarized mitoEV), FCCP-treated EV were considered almost fully depolarized and 5 % of them with the highest MitoTracker Red signal intensity were selected as polarized. Using this strategy, C-EV showed to contain about 70 % polarized mitoEV relative to total EV (Fig. 3C) suggesting that most C-mitoEV carry respiring mitochondria. Taken together, this data showed that C-mitoEV possess a functional proton gradient that can be inhibited by FCCP, and FCCP treatment can be used to determine the percentage of polarized mitoEV.

### 3.3. Donor EC mitochondrial redox determines mitoEV numbers and mitochondrial cargo quality

**Fig. 4.**
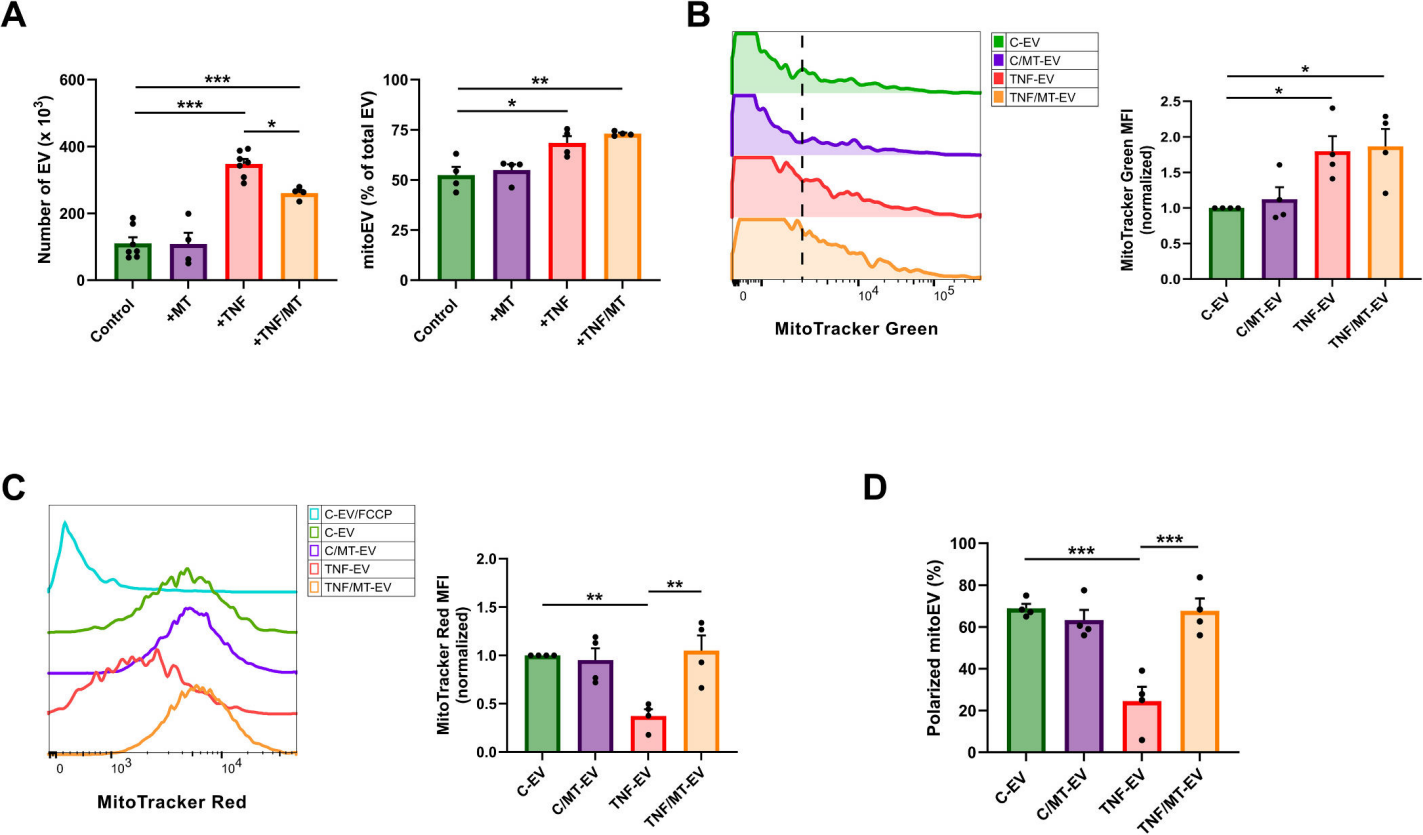
The donor EC mitochondrial redox state determines (mito)EV release kinetics and the mitoEV mitochondrial cargo polarization state. (A) EC treatment with TNF-α significantly increased the release rate (plotted as number of EV×10^2^ per 10^5^ EC at 24 h) of EV and mitoEV (plotted as percentage of total EV) compared to untreated EC. Cotreatment with MT (1 μM, 24 h plus a 30 min preincubation) of EC stimulated with TNF-α (10 ng/mL, 24 h) decreased the production rate of total EV, without changing the percentage of released mitoEV. (B) MT cotreatment did not affect the presence of the larger mitochondrial cargo in TNF-EV. (C) MT cotreatment significantly improved the mitochondrial cargo polarization of TNF-EV, but had no effect on the mitochondrial cargo polarization of C-EV. (D) MT cotreatment significantly increased the number of TNF-mitoEV carrying polarized mitochondria (plotted as % of total EV) compared to that in TNF-EV (without MT cotreatment). Data are plotted as mean±SE of n = 4 independent experiments. *, P ≤ 0.05; **, P ≤ 0.01; ***, P ≤ 0.001 compared to either EC, C-EV, or TNF-EV.

EC stimulation with TNF-α (10 ng/mL, 24 h) significantly increased the number of EV (TNF-EV) released from 10^5^ EC, as well as the proportion of mitoEV to about 70 % of total EV, compared to C-EV and C-mitoEV, respectively (Fig. 4A). Cotreatment with the mitochondria-targeted antioxidant MT (1 μM, 30 min preincubation followed by 24 h) significantly inhibited the TNF-induced increase in released EV (TNF/MT-EV), with no significant effect on the percentage of released mitoEV (Fig. 4A). MT had no effect on the release rate of C-EV or the percentage of C-mitoEV (Fig. 4A). When EV were stained with MitoTracker Green to assess mitochondrial cargo mass, frequency histograms and MFI measurements showed that TNF-EV carried a significantly larger mitochondrial cargo mass compared to C-EV and C/MT-EV (Fig. 4B). MitoTracker Green MFI was comparable between TNF-EV and TNF/MT-EV suggesting that MT does not affect the TNF-induced increase in mitochondrial cargo mass (Fig. 4B). In summary, TNF causes the release of significantly more mitoEV with a larger mitochondrial load compared to C-EV, and MT inhibits the TNF-induced increase in mitoEV number, but not the increase in mitochondrial load.

MitoTracker Red MFI, an indicator of mitoEV ΔΨ_m_ (Fig. 3A-B), was significantly reduced in TNF-EV compared to C-EV (Fig. 4C). MitoTracker Red MFI in TNF/MT-EV was restored to levels comparable to C-EV and C/MT-EV suggesting that cotreatment of donor EC with MT can block the depolarization of mitochondrial cargo in TNF-EV (Fig. 4C). When gating was applied to quantify the polarized mitoEV, MT cotreatment was found to restore the percentage of polarized mitoEV (relative to total EV) to those levels of polarized mitoEV released from control EC, either untreated or treated with MT (Fig. 4D). MT treatment alone (without TNF) had no effect on the polarization status of C-mitoEV. Together, these findings showed that, while inflammatory stimuli increase mitoEV abundance and mitochondrial cargo mass, they also compromise mitochondrial cargo quality/polarization. Importantly, treatment of donor EC with mitochondria-targeted antioxidants, such as MT, can preserve the ΔΨ_m_ of the mitoEV cargo.

### 3.4. MitoEV mitochondrial cargo localizes to target EC mitochondria and regulates cell function

**Fig. 5.**
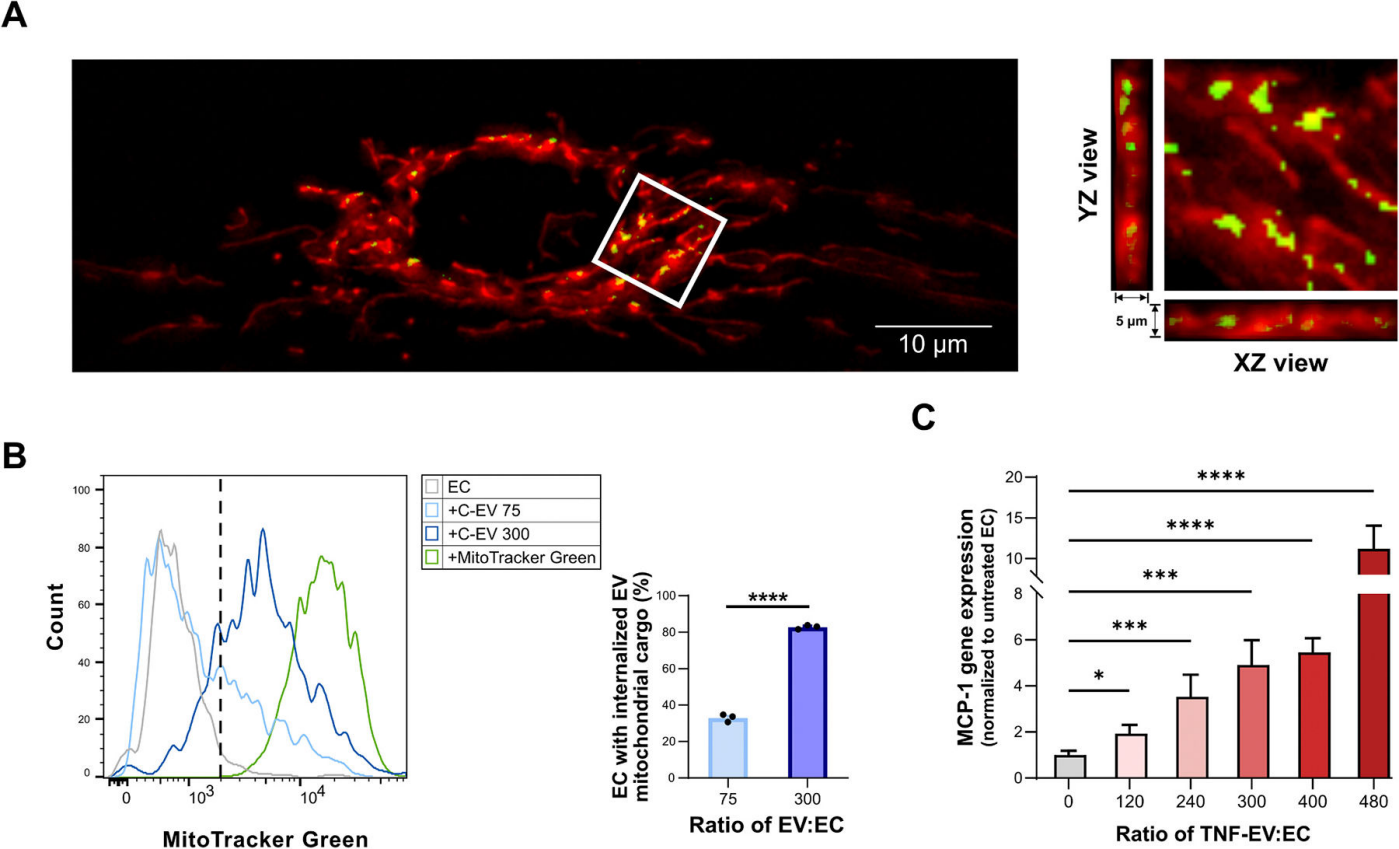
EC-mitoEV donate their mitochondrial cargo to target EC and regulate gene transcription. (A) Donor control EC were stained with MitoTracker Green and cultured for an additional 24 h prior to media collection/EV isolation. EV were incubated for 24 h with target naïve EC transduced to express mitochondria-RFP. The image on the left shows maximum projection of z-stacks consisting of 13 images at a 0.4 μm step covering the 5 μm-thick mitochondrial network of a target EC. A selected region of interest (R0I; 7.5 ×7.5 μm) outlined by a white box shows locations of overlap between red and green fluorescence. In the image on the right, XZ and YZ projections (different scale than the original R0I) confirmed that mitoEV-donated mitochondria colocalize with the 3D space of the target EC mitochondrial network. (B) Target naïve EC were incubated with two different doses of EV from MitoTracker Green-stained donor EC for 24 h. A representative histogram of target EC fluorescence, due to uptake of the mitoEV mitochondrial cargo, is shown. Data are plotted as mean±SE of n = 3 independent experiments. ****, P ≤ 0.0001 compared to the lower EV dose. (C) Incubation of target naïve EC with TNF-EV for 24 h dose-dependently increased their MCP-1 gene expression. Data are plotted as mean±SE of n = 5 independent experiments. *, P ≤ 0.05; **, P ≤ 0.01; ***, P ≤ 0.001; and ****, P ≤ 0.0001 compared to target EC not incubated with EV.

To assess the location of donated mitoEV mitochondria inside target EC, EV from donor control EC previously stained with MitoTracker Green were incubated for 24 h with target naïve EC transduced to express mitochondria-targeted RFP, and target EC were observed using fluorescence microscopy. Mitochondria donated from mitoEV were found to colocalize with target EC mitochondria: The maximum projection (left) and, especially the orthogonal views of z-stacks (right), confirmed the incorporation of donated mitochondria within the 3D space of the target EC mitochondrial network (Fig. 5A). Colocalization occurred independently of the donor EC activation state, for either C-EV (Fig. 5A) or TNF-EV (colocalization images with TNF-EV are not shown). Flow cytometry was used to quantitatively assess EV-mediated mitochondrial transfer: EC incubated with increasing concentrations of C-EV (ratio of EV:EC of either 75 or 300) for 24 h showed a dose-dependent uptake of mitochondrial cargo with over 80 % of recipient EC becoming MitoTracker Green^+^ at the higher EV dose (Fig. 5B). To evaluate the consequences of EV-mediated mitochondrial transfer on target EC function, naïve EC were incubated with TNF-EV across a range of concentrations for 24 h. A significant and dose-dependent increase in MCP-1 gene expression was observed (Fig. 5C) suggesting that TNF-EV, but not C-EV, can elicit proinflammatory signaling in target EC. Taken together, this data showed that robust mitochondrial uptake and downstream events occur following a 24 h incubation of ≥ 240 EV per target EC (Fig. 5B-C).

### 3.5. EV-induced paracrine effects are regulated by the quality of mitoEV-donated mitochondria

**Fig. 6.**
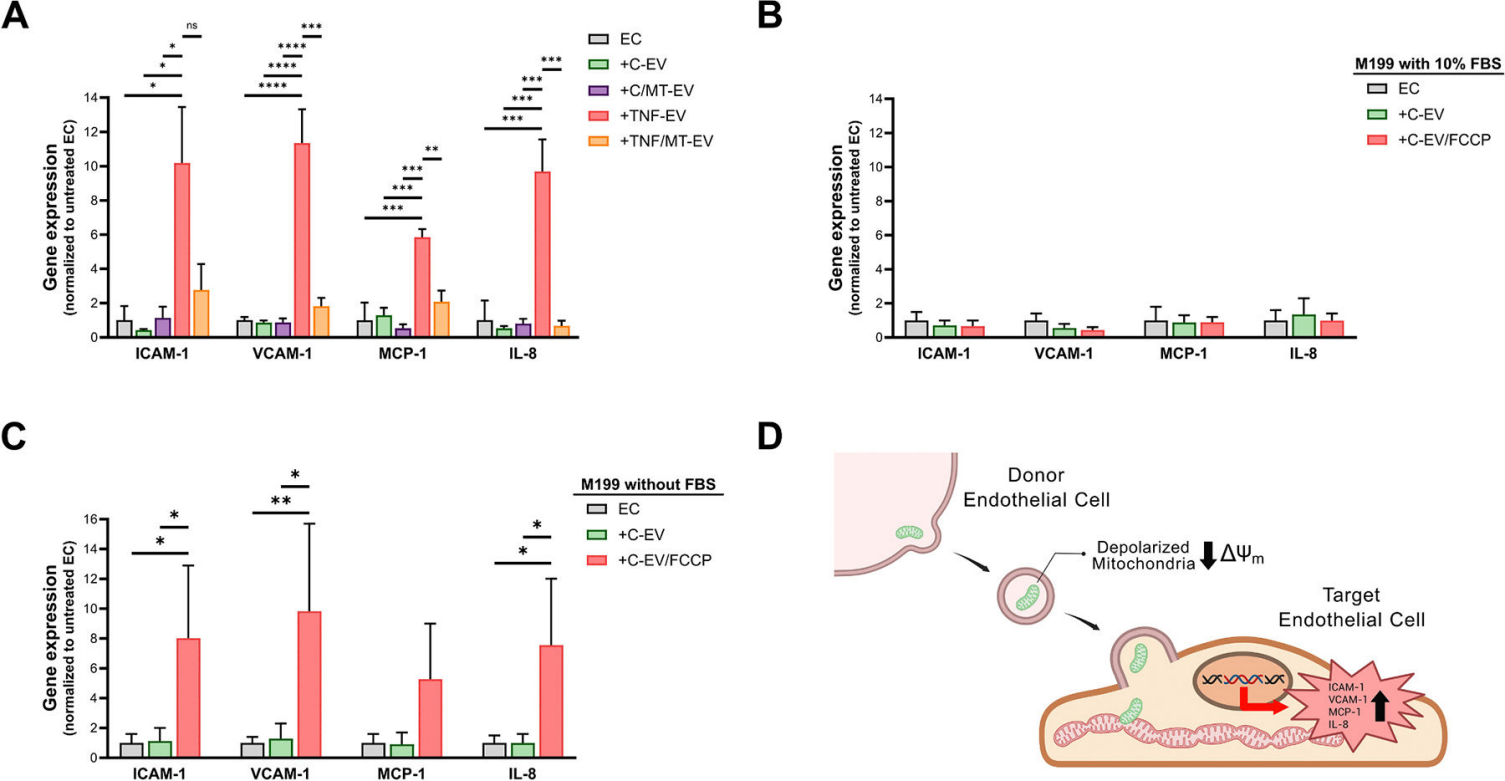
The EV paracrine effects are determined, at least in part, by their mitoEV mitochondrial cargo polarization state. (A) While TNF-EV caused a significant upregulation of inflammatory gene expression in target naïve EC, TNF/MT-EV lost their paracrine inflammatory capacity (gene expression was at levels similar to those in EC either not incubated with EV, incubated with C-EV, or incubated with C/MT-EV). Data are plotted as mean±SE of n = 3 independent experiments. *, P ≤ 0.05; **, P ≤ 0.01; ***, P ≤ 0.001; and ****, P ≤ 0.0001 compared to indicated respective groups. (B) Target naïve EC incubation with C-EV/FCCP (ratio of EV:EC of 600) in M199 with 10 % FBS did not trigger any significant inflammatory gene expression compared to that in EC either not incubated with EV or incubated with C-EV. Data are plotted as mean±SE of n = 3 independent experiments. (C) Target naïve EC incubation with C-EV/FCCP (ratio of EV:EC of 600) in M199 without FBS significantly increased inflammatory gene expression compared to that in EC either not incubated with EV or incubated with C-EV. Data are plotted as mean±SE of n = 6 independent experiments. *, P ≤ 0.05; **, P ≤ 0.01 compared to EC either not incubated with EV or incubated with C-EV. (D) Based on findings in the present study, a model of horizontal mitochondrial transfer is proposed where donation of mitochondrial cargo with decreased/lost ΔΨ_m_ from EC-mitoEV to target EC results in paracrine inflammatory signaling. Created in BioRender. Manzar, Z. (2025) https://biorender.com/5qeeoii .

To assess whether the TNF-mitoEV mitochondrial cargo quality plays a causative role in the EV-induced paracrine inflammatory signaling, target naïve EC were incubated for 24 h with either C-EV, C/MT-EV, TNF-EV, or TNF/MT-EV, and assayed for inflammatory gene expression via RT-qPCR. EC incubation with either C- or C/MT-EV had no significant effect on ICAM-1, VCAM-1, MCP-1, and IL-8 transcript levels, whereas TNF-EV significantly upregulated all genes tested confirming the TNF-EV ability to induce inflammation in recipient EC (Fig. 6A). In contrast, TNF/MT-EV failed to induce inflammatory gene expression suggesting a potential link between the mitoEV mitochondrial cargo quality and EV paracrine effects. However, EC treatment with MT is expected to broadly alter EV cargo composition (nucleic acids, proteins, and lipids), thereby many factors may have contributed towards the loss of proinflammatory capacity by TNF/MT-EV. To test whether the mitoEV mitochondrial cargo depolarization on its own is sufficient to elicit inflammatory responses in target EC, the C-EV pellet was resuspended in either complete M199 medium (with 10 % FBS) or M199 medium without FBS and treated with FCCP (2.5 μM, overnight), and the produced C-EV/FCCP were washed, resuspended in their respective media, and incubated with target naïve EC in their respective media for 24 h. The reason for repeating the experiment in M199 medium without FBS is because mitochondrial respiration is known to recover when cells (in our case, mitoEV) are maintained in serum-containing, but not serum-free, media over a period of several hours [32,33]. When target naïve EC were incubated with C-EV/FCCP (EV:EC ratio of either 300 or 600) in complete M199 medium, there was no increase in inflammatory gene expression compared to that in either vehicle-treated EC or EC incubated with C-EV (Fig. 6B; data shown are from EV:EC ratio of 600). However, when target naïve EC were incubated with C-EV/FCCP (EV:EC ratio of 600) in M199 medium without FBS, there was a significant upregulation of inflammatory gene expression compared to that in either vehicle-treated EC or EC incubated with C-EV at the same EV:EC ratio (Fig. 6C). When target EC were incubated with C-EV/FCCP at EV: EC ratio of 300 in M199 medium without FBS, gene expression levels did not reach significant difference (not shown). Take together, these results suggest that a decrease/loss of mitoEV ΔΨ_m_ on its own is sufficient to activate inflammatory signaling in target EC (Fig. 6D), and may also play a role in the paracrine inflammatory response induced by TNF-EV.

## 4. Discussion

Our findings revealed that a) cultured human EC release lEV and, under either control or activated conditions, a significant proportion of them are mitoEV, b) most of C-mitoEV contain polarized mitochondria, whereas most of TNF-mitoEV contain depolarized mitochondria, c) the mitochondrial redox state of donor EC determines whether donor EC will release polarized or depolarized mitoEV, as well as the release kinetics of (mito)EV, d) mitoEV mitochondrial cargo polarization negatively correlates with the EV ability to cause inflammatory signaling in target naïve EC, and e) mitoEV mitochondrial cargo depolarization on its own is sufficient to trigger paracrine inflammatory signaling. These findings suggest that protecting the mitoEV mitochondrial cargo quality, as assessed by the cargo ΔΨ_m_, may, at least in part, prevent the propagation of EC inflammation/dysfunction and development of CVD. To the best of our knowledge, most studies have modulated EV function indirectly, either by treating cultured cells, including EC, with cytokines, metabolic stressors, and/or antioxidants and isolating EV or by isolating circulating EC-EV from blood samples, and then studying the EV effects on cultured target EC [10,18,34–36]. Although several of those studies associated the EV-mediated transfer of dysfunctional mitochondria with target EC inflammation [10,18,34], our study is the first to identify the mitochondrial cargo ΔΨ_m_ as a determinant factor in paracrine inflammatory signaling. This was done by using FCCP to specifically depolarize the mitoEV mitochondrial cargo, without changing mitochondrial cargo mass or any other cargo component, and showing that the mitoEV-mediated transfer of depolarized mitochondria is sufficient to trigger inflammation.

MitoEV are known to trigger inflammation because of mitochondrial cargo components, such as mtDNA, mtRNA, mROS, ATP, cardiolipin, and Ca^2+^, that act as mitochondrial damage-associated molecular patterns (mtDAMP) [37]. Mitochondrial nucleic acids, especially when oxidized by mROS, are effective proinflammatory stimuli [38]. Via opening of the mitochondrial permeability transition pore (mPTP) in the mitochondrial inner membrane and a compromise of the outer membrane (the latter may not be necessary, depending on the specific mtDAMP), mtDAMP can be released from mitoEV-donated mitochondria into the cytosol of target cells, where they activate the inflammasome and nuclear factor (NF)-κB signaling leading to production of proinflammatory cytokines [37]. That mechanism would explain the effects caused by the mitoEV portion of TNF-EV on target naïve EC. Importantly, TNF-EV, independently of whether they carry mitochondria, are known to differ in nucleic acid and protein cargo from C-EV, and these cargo components may be responsible for paracrine inflammatory signaling [31,39]. In the case of C-EV/FCCP, FCCP treatment is expected to uncouple the C-EV mitochondrial cargo and lower mROS production, but, once these mitochondria are transferred inside target cells, they are expected to also release various mtDAMP and initiate inflammatory signaling. C-EV/FCCP do not contain the nucleic acids and proteins that TNF-EV have, and their mtDAMP may not involve oxidized nucleic acids, which may explain the decreased capacity of C-EV/FCCP to induce paracrine inflammation compared to that caused by the same concentration of TNF-EV.

Published work by others supports the idea that mitoEV released from stressed cells, including EC, via their dysfunctional mitochondrial cargo and associated mtDAMP, can induce EC inflammation: Specifically, LPS-activated monocytes were shown to release EV enriched in mtDAMP, such as oxidized mtRNA; these EV caused inflammatory responses in target naïve EC. In agreement with the present study, when the donor cells’ mitochondrial respiration was protected by antioxidants, EV failed to trigger EC inflammation [10]. Similarly, EV from LPS- or oligomycin-treated EC were found to have decreased expression of selective mitochondrial electron transport chain (ETC) proteins, decreased ΔΨ_m_, and increased mROS levels compared to EV from control EC; these EV induced ROS-mediated inflammatory signaling in target naïve EC [18]. Last, EC exposure to hyperglycemic conditions led to release of EV with increased NADPH oxidase activity/ROS levels, and these EV exhibited proinflammatory properties [34]. Mechanisms that cause EC dysfunction are particularly important in atherosclerosis where EC inflammation drives the first step of monocyte adhesion and transendothelial migration, prior to monocyte differentiation into macrophages followed by conversion into foam cells and plaque formation [40]. It is noteworthy that EV derived from various types of activated donor cells, besides triggering inflammation and, in some case, EC death signaling, they were also shown to upregulate adaptive antioxidant responses in target EC [41,42].

This study also showed that C-EV, and, in general, EV with a polarized mitoEV subset, are unable to induce paracrine inflammatory responses. Furthermore, EC cotreatment with TNF-α and MT was found to mitigate the TNF-induced activation of donor EC, lead to production of mitoEV with restored mitochondrial quality/polarization, and abolish the TNF-EV ability to trigger inflammatory responses in target EC, highlighting mitoEV ΔΨ_m_ as a potential diagnostic and prognostic biomarker of EC dysfunction. Published in vitro and in vivo work supports a cytoprotective role for C-EV on EC function: EV from control brain EC significantly increased ATP levels, OCR, and extracellular acidification rate (ECAR), a measure of glycolytic activity, in an in vitro model of ischemic brain EC [15]. In a murine middle cerebral artery occlusion model of ischemia/reperfusion injury, EC-EV administration reduced brain infarct size and improved post-stroke behavioral outcomes compared to untreated controls [43,44]. Interestingly, Peruzzotti-Jametti et al. showed that neural stem cell-derived EV have the capacity to increase OCR and ECAR, and reduce the expression of proinflammatory markers in LPS-stimulated macrophages, but the EV failed to induce these regenerative effects when pretreated with FCCP [45]. The latter study together with our study suggest that the mitoEV ΔΨ_m_ may help explain not only the harmful effects of depolarized mitoEV that deliver mtDAMP and initiate inflammatory pathways, but also the beneficial effects of polarized mitoEV that bolster target cell mitochondrial respiration and metabolism.

Imaging flow cytometry was employed in this study for visual confirmation of mitoEV staining, and measurements of mitoEV count, area, and mitochondrial cargo mass and polarization. Single-vesicle detection using the ImageStream^X^ platform was shown by others to be a powerful tool for characterization of EV from various cells/tissues [46–48]. Indeed, the Amnis ImageStream^X^ MkII-401 was capable of detecting and phenotyping particles with diameter as small as 20–100 nm, far below the resolution limit of conventional flow cytometers, while requiring minimal sample preparation and enabling multiparametric studies. The BD LSRFortessa X-20, employed for EC flow cytometry, was unable to distinguish mitoEV from background signal and yielded large variations when measuring replicates confirming prior reports that imaging flow cytometry is required for robust, reproducible EV analysis [47]. While others used JC-1 or TMRM [18,49], we verified the presence of respiring mitoEV mitochondrial cargo using the MitoTracker Red dye and showed that it responds to treatment with increasing FCCP concentrations without any effects on MitoTracker Green staining. This demonstrated that the FCCP treatment depolarizes the mitoEV mitochondrial cargo without impacting the mitochondrial cargo mass. To study mitoEV uptake by target EC, two methods were employed: Flow cytometry for quantification and fluorescence microscopy for visual confirmation. Flow cytometry showed a dose-dependent EV uptake with over 80 % of target EC becoming MitoTracker Green^+^ at a ratio of 300 EV:EC and a 24 h incubation. Following dual labeling with MitoTracker Green (to stain the donor EC mitochondria prior to their packaging inside mitoEV) and a mitochondria-targeted RFP viral construct (to label the target EC mitochondria), fluorescence microscopy revealed the green signal from mitoEV mitochondria to colocalize within the 3D space of the red mitochondrial network of target EC. Dual labeling with different MitoTracker dyes (despite the fact that some of them are known to be ΔΨ_m_-dependent) and/or viral vectors to tag mitochondrial proteins has been used previously to demonstrate colocalization of mitoEV-donated mitochondria with the recipient cell mitochondria [15,17,43]. All the paracrine effects shown by us and others may be attributed, at least in part, to the interactions of the mitoEV mitochondrial cargo with the target cell’s mitochondrial network. Not all donated mitochondria reach the mitochondrial network, based on reports that the EV cargo can be shuttled to lysosomes for degradation [50,51].

Since the average length of EC mitochondria is ~1 μm [12,13] and our NTA and TEM analyses showed the upper limit of EV size to be ~1 μm, it is expected that a percentage of EV, at least the larger EV, can carry whole mitochondria. Our TEM analysis showed mitoEV as small as 200 nm carrying a single mitochondrion suggesting that these may be mitochondrial fragments. Twig et al. [52] published that each mitochondrial fission event produces two fragments, one that is polarized and another one that is depolarized, and the fragments that are polarized can undergo fusion leaving out the depolarized ones. Under persistent oxidative stress, the process of fission continues until all fragments become depolarized and are not able to undergo fusion anymore [52,53]. In our work, we used MitoTracker Green that binds to cysteines of mitochondrial proteins independently of ΔΨ_m_, and MitoTracker Red that binds to mitochondrial proteins of only the polarized mitochondria, and showed that treatment of C-EV with the protonophore FCCP results in loss of the MitoTracker Red, but not MitoTracker Green, fluorescence. Hence, it is probable that, besides whole mitochondria, mitochondrial fragments are encapsulated within some of the C-EV and TNF-EV and those fragments may have membrane potential, and, hence, they will bind to MitoTracker Red, besides MitoTracker Green. Our data also showed the mitochondrial cargo in TNF-EV to have on average a lower membrane potential compared to the membrane potential found in C-EV.

There are several limitations in the present study: All experiments were conducted using cultured EC and EC-EV isolates from conditioned media. While EC-EV have been isolated from blood samples of patients and respective controls, and the EC-EV effects have been studied on cultured EC [35,36], there has been no systematic analysis of the mitoEV mitochondrial cargo ΔΨ_m_. In vivo validation studies are needed, including guidelines on storage conditions to help maintain mitochondrial cargo polarization/respiration following EV harvesting. Furthermore, in vitro studies do not replicate the dynamic interactions among EV released from different cell types, including circulating cells, and the vascular endothelium (e.g., EV opsonization and uptake by immune cells, etc.) that may influence the numbers and activity/downstream effects of EC-mitoEV. Second, while the evidence suggests that ΔΨ_m_ is a key determinant of mitoEV function, this study did not define the specific mtDAMP that may be enriched in TNF-EV, such as mROS, oxidized mtDNA or mtRNA, and may be responsible for driving paracrine signaling. Third, use of a ΔΨ_m_-sensitive dye followed by imaging flow cytometry has inherent limitations, since it relies on fluorescence surrogates for mitochondrial functionality. Although functional assays were not performed in this study, others have measured ATP levels and OCR in EC-EV and have found them to be significantly lower in EV from LPS- or oligomycin-treated EC compared to those in C-EV [18]. Finally, the inherent EV heterogeneity in size and cargo, and lack of standardization and established EV-specific protocols pose challenges [54]. The present study focused on lEV, but our samples contain a small percentage of sEV (100–150 nm in diameter), and a spectrum of mitochondrial (as shown by TEM), as well as other, components is expected across different EV sizes.

The discovery of a causative role of mitoEV mitochondrial cargo depolarization in paracrine inflammatory signaling has potential clinical and therapeutic applications. Circulating EC-EV with a high load of non-respiring mitochondria (low ΔΨ_m_) may be a better biomarker of EC dysfunction in cardiometabolic disorders and CVD, and may even predict adverse clinical outcomes. Therapeutic strategies may need to focus on reducing the production of depolarized mitoEV by improving mitochondrial function (using mitochondria-targeted antioxidants or sirtuin activators) and/or enhancing mitophagy (using parkin activators) in donor cells/tissues. Last, there is burgeoning interest in utilizing native EV or EV-like particles as delivery vehicles of mitochondria [55]. Any such approaches would benefit from a better understanding of mitochondrial biology to ensure the delivered organelles have maintained their ΔΨ_m_.

## 5. Conclusions

The present work demonstrated that EC-mitoEV donate their mitochondrial cargo to target EC, and the donated mitochondria incorporate themselves into the target EC mitochondrial network. The mitochondrial redox state of donor EC determined both the quality/polarization of the mitoEV mitochondrial cargo, as well as the proportion of polarized mitoEV. Both of these parameters were inversely correlated with the capacity of TNF-EV to induce inflammatory signaling in target EC suggesting a mechanistic link. In agreement to that, C-EV treated with FCCP were capable of inducing a paracrine inflammatory response, although the response required a higher dose of C-EV/FCCP compared to TNF-EV. In conclusion, mitoEV ΔΨ_m_ may, at least in part, determine the EC-EV paracrine effects, and measuring mitoEV ΔΨ_m_ may prove useful in predicting the downstream effects of EV. Mitoprotective pharmacological interventions on either donor EC or circulating EC-mitoEV could open new avenues for mitigating vascular inflammation/dysfunction and CVD development.

## Data Availability

Data will be made available on request.

## Abbreviations:

EV: Extracellular vesicles
EC: Endothelial cells
EC-EV: Endothelial cell-derived EV
CVD: Cardiovascular diseases
MitoEV: Mitochondria-carrying EV
TNF-α: Tumor necrosis factor-α
TNF-EV: EV Derived from TNF-α-treated EC
C-EV: EV Derived from control EC
FCCP: Carbonyl cyanide p-trifluoromethoxyphenylhydrazone
ΔΨ_m_: Mitochondrial membrane potential
lEV: Large-size EV
sEV: Small-size EV
iCM: Induced pluripotent stem cell-derived cardiomyocytes
MSC: Mesenchymal stem cells
ROS: Reactive oxygen species
OGD: Oxygen-glucose-deprived
OCR: Oxygen consumption rate
LPS: Lipopolysaccharide
MROS: Mitochondrial ROS
FITC: Fluorescein isothiocyanate
APC: Allophycocyanin
RBC: Red blood cells
HUVEC: Human umbilical vein EC
PBS: Phosphate buffered saline
MT: MitoTEMPO
PS: Phosphatidylserine
dEV: EV Devoid of mitochondrial cargo
NucDNA: Nuclear DNA
MtDNA: Mitochondrial DNA
β2M: β−2-microglobulin
CYTB: Cytochrome
MT-TL1: Mitochondrial tRNA-Leu UUA/G 1
ND1: NADH dehydrogenase 1
ICAM-1: Intercellular adhesion molecule-1
VCAM-1: Vascular cell adhesion molecule-1
MCP-1: Monocyte chemoattractant protein-1
IL-8: Interleukin-8
GAPDH: Glyceraldehyde-3-phosphate dehydrogenase
ACTB: β-actin
ANOVA: Analysis of variance
MtDAMP: Mitochondrial damage-associated molecular patterns
mPTP: Mitochondrial permeability transition pore
NF-κB: Nuclear factor-κB
ETC: Electron transport chain
ECAR: Extracellular acidification rate
